# Parallel Evolution in *Streptococcus pneumoniae* Biofilms

**DOI:** 10.1093/gbe/evw072

**Published:** 2016-04-15

**Authors:** Nicholas W. V. Churton, Raju V. Misra, Robert P. Howlin, Raymond N. Allan, Johanna Jefferies, Saul N. Faust, Saheer E. Gharbia, Richard J. Edwards, Stuart C. Clarke, Jeremy S. Webb

**Affiliations:** ^1^Centre for Biological Sciences, Faculty of Natural and Environmental Sciences, University of Southampton, United Kingdom; ^2^Academic Unit of Clinical and Experimental Sciences, Faculty of Medicine, University of Southampton, United Kingdom; ^3^Institute for Life Sciences, Faculty of Natural and Environmental Sciences, University of Southampton, United Kingdom; ^4^Genomics Research Unit, Microbiology Services, Public Health England, Colindale, United Kingdom; ^5^NIHR Southampton Respiratory Biomedical Research Unit, Southampton, United Kingdom; ^6^Southampton NIHR Wellcome Trust Clinical Research Facility, University Hospital Southampton NHS Foundation Trust, United Kingdom; ^7^Public Health England, Southampton, United Kingdom; ^8^School of Biotechnology and Biomolecular Sciences, University of New South Wales, Sydney, Australia

**Keywords:** biofilm, *Streptococcus pneumoniae*, genomic diversification, parallel evolution

## Abstract

*Streptococcus pneumoniae* is a commensal human pathogen and the causative agent of various invasive and noninvasive diseases. Carriage of the pneumococcus in the nasopharynx is thought to be mediated by biofilm formation, an environment where isogenic populations frequently give rise to morphological colony variants, including small colony variant (SCV) phenotypes. We employed metabolic characterization and whole-genome sequencing of biofilm-derived *S. pneumoniae* serotype 22F pneumococcal SCVs to investigate diversification during biofilm formation. Phenotypic profiling revealed that SCVs exhibit reduced growth rates, reduced capsule expression, altered metabolic profiles, and increased biofilm formation compared to the ancestral strain. Whole-genome sequencing of 12 SCVs from independent biofilm experiments revealed that all SCVs studied had mutations within the DNA-directed RNA polymerase delta subunit (RpoE). Mutations included four large-scale deletions ranging from 51 to 264 bp, one insertion resulting in a coding frameshift, and seven nonsense single-nucleotide substitutions that result in a truncated gene product. This work links mutations in the *rpo*E gene to SCV formation and enhanced biofilm development in *S. pneumoniae* and therefore may have important implications for colonization, carriage, and persistence of the organism. Furthermore, recurrent mutation of the pneumococcal *rpo*E gene presents an unprecedented level of parallel evolution in pneumococcal biofilm development.

## Introduction

*Streptococcus pneumoniae* is an encapsulated bacterium that forms part of the human respiratory biota (Tocheva et al. 2011). Carriage is asymptomatic but known to facilitate transmission of the pathogen and precede pneumococcal infection (Simell et al. 2012). The resultant diseases can range from acute otitis media and pneumonia to invasive disease including septicemia and meningitis. Although much disease is preventable by vaccination (Gladstone et al. 2011), it is still a major cause of morbidity and mortality worldwide (O'Brien et al. 2009) requiring continuous surveillance.

Carriage of the pneumococcus is thought to be facilitated by biofilm formation (Marks et al. 2012); biofilms are complex aggregations of bacteria which adhere to inert or living surfaces and are encased in an extracellular polymeric substance (Costerton et al. 1995). Biofilms are a major contributor to chronic disease (Hall-Stoodley and Stoodley 2009), and it is estimated that around 65% of infections are a result of biofilm formation (Potera 1999). Furthermore, pneumococcal biofilm formation has been directly observed in chronic pneumococcal infections (Hall-Stoodley et al. 2006; Nistico et al. 2011).

Microorganisms have been used extensively to study rapid evolutionary changes in real time (Buckling et al. 2009); large populations, short generation times, and relatively simple genomes, coupled with the advent of next-generation whole-genome sequencing, allow us to address fundamentally important questions about adaption, mutation, and morphological change within bacterial populations. Growth of respiratory pathogens within biofilms can result in rapid genetic diversification; colony morphology variants can emerge that exhibit increased adhesion, dispersal, and/or recalcitrance to oxidative stress and antibiotics (Boles et al. 2004; Webb et al. 2004; Allegrucci and Sauer 2008; Mitchell et al. 2010). Studies of pneumococcal biofilms have shown colony variants to be both phenotypically and genetically distinct from ancestral strains (Waite et al. 2001; Allegrucci and Sauer 2007, 2008; Domenech et al. 2009). *Streptococcus pneumoniae* is a highly recombinant organism and prone to mutations that facilitate long-term persistence in the host (Croucher et al. 2011). This phenomenon of biofilm-mediated genetic diversification is thought to be a key factor in pneumococcal persistence within the host and is thought to act as a survival strategy, whereby species with the highest genetic diversity have the greatest ability to withstand perturbations within the environment (Boles et al. 2004). The mechanisms by which bacteria attain such diversity remain unclear and are fundamental to understanding how biofilms persist despite continual antimicrobial therapy and is potentially relevant to future vaccine design (Jefferies et al. 2011).

Cells growing within a biofilm are subjected to steep oxygen and pH gradients (Debeer et al. 1994; Sternberg et al. 1999), resulting in chemical gradients and microenvironmental niches within the biofilms (Debeer et al. 1994). Adaptation to changes in environmental conditions is essential for survival within the biofilm (Ehrlich et al. 2005). Recently, spontaneous mucoid variants have been shown to repeatedly arise in *Pseudomonas fluorescens* colonies (Kim et al. 2014). These variants were shown to spatially position themselves on the surface of the colony to optimize access to oxygen and nutrients. The authors proposed that the mucoid variants underwent strong selection in mixed colony experiments where rapid increase in the mucoid phenotype resulted in dominance over the wild-type (WT) strain (Kim et al. 2014). Furthermore, whole-genome sequencing of over 500 mucoid variants revealed a striking example of parallel evolution at the *rsm*E locus (Kim et al. 2014). These data suggest that parallel evolution can underlie diversification in surface-attached communities of bacteria within biofilms.

In this work, we use a clinical isolate of serotype 22F, which was chosen as a model serotype because its prevalence in pneumococcal carriage and disease has increased in both the United Kingdom and the United States since the implementation of pneumococcal conjugate vaccines (Jacobs et al. 2008; Tocheva et al. 2011). We perform both conventional phenotyping and next-generation sequencing to characterize the genetic diversity that can arise from biofilm-derived pneumococci. Mutations that arise in serotype 22F pneumococcal variants were identified and related to phenotypic characteristics. We assess genotype–phenotype relationships and provide insight into genomic factors that are strongly favored under natural selection in pneumococcal biofilms.

## Results

**Fig. 1.—:**
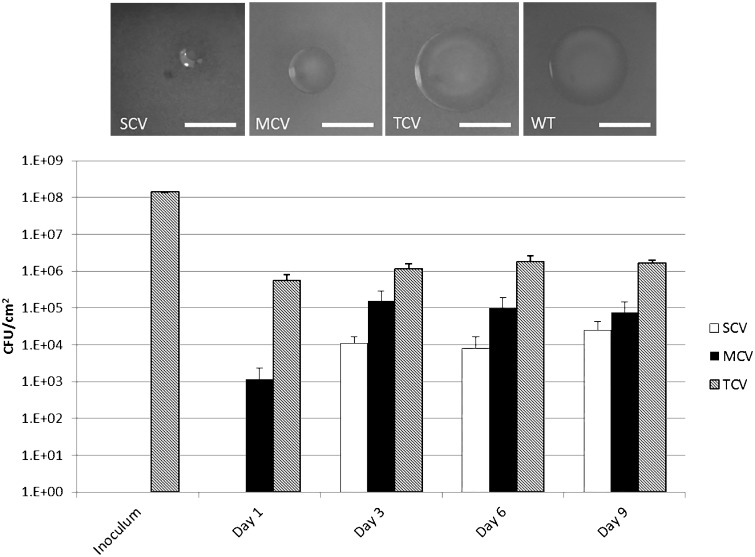
CFU enumeration of biofilm-derived pneumococcal variants. Cells were harvested from triplicate pneumococcal biofilms from two independent experiments at time points 1, 3, 6, and 9 days and plated onto CBA to assess for changes in colony morphology. Variants were defined as follows: SCVs (<0.5 mm), MCVs (0.5–1 mm), and TCVs (>1 mm). Data represent the mean CFU counts from the two biofilm experiment. Error bars represent standard error. Images of colonies were taken on CBA under ×6 objective on a Leica dissection Stereomicroscope. Scale bar = 1 mm.

**Fig. 2.—:**
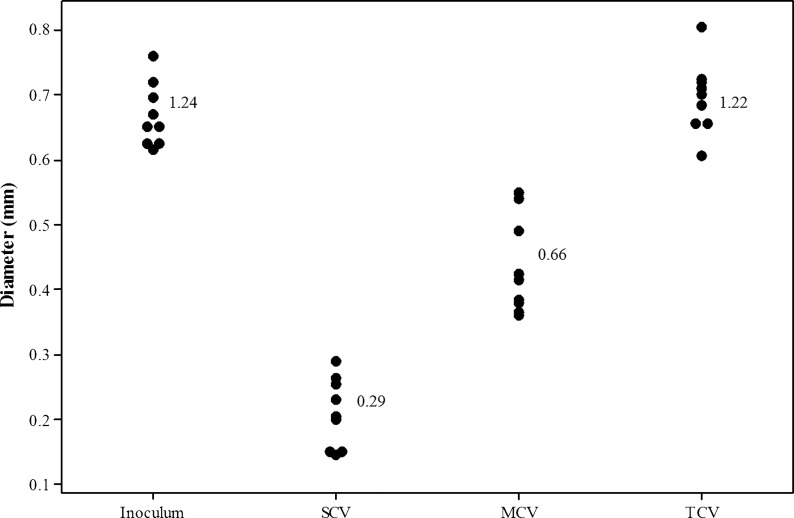
Colony quantification of serotype 22F biofilm-derived colony variants. Diameter values of the three distinct biofilm-derived colony variant populations harvested from pneumococcal biofilms quantified using ImageJ analysis software. A total of nine colony variants from triplicate biofilms were measured. Numbers signify mean values.

Three distinct *S. pneumoniae* colony variants were observed after 3 days of biofilm growth in microtitre plates: small colony variants (SCVs) (<0.5 mm), medium colony variants (MCVs) (0.5–1 mm), and typical colony variants (TCVs) (>1 mm) (fig. 1). Consistent with the ancestral strain, all variants stained Gram positive, exhibited alpha hemolysis, and remained optochin sensitive. Colony-forming unit (CFU) enumeration of the biofilm biomass showed that the majority of colonies formed retained the WT (TCV) morphology, whereas SCVs and MCVs represented approximately 1% and 4% of the population, respectively, by day 9 (fig. 1). Quantification of the colony diameter confirmed that SCVs and MCVs were significantly distinct from each other and the WT ancestral strain (WT vs. MCV: *T* = 13.51, df = 16, *P* < 0.001; WT vs. SCV: *T* = 21.38, df = 16, *P* < 0.001; MCV vs. SCV: *T* = 9.73 df = 16, *P* < 0.001). In contrast, colonies with the TCV phenotype did not differ significantly from the WT ancestral strain (*T* = 0.28, df = 16, *P* > 0.05) (fig. 2). All variant morphologies had smooth margins. To determine whether this variation in morphology was a direct result of biofilm growth and not simply prolonged growth, planktonic WT cultures were grown for 9 days, subculturing daily into fresh brain heart infusion (BHI) to maintain cell viability. At days 1, 3, 6, and 9, cells were serially diluted and plated onto blood agar to assess morphology. Over the 9-day time course, the WT morphology remained consistent and the presence of small and medium sized colonies was not observed (data not shown).

**Fig. 3.—:**
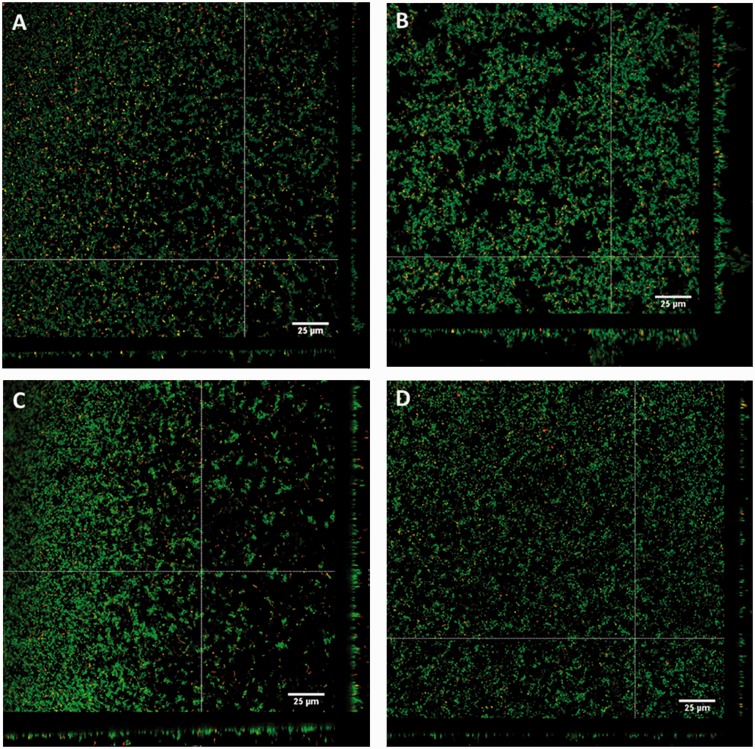
Biofilm formation of the biofilm-derived colony variants. Three-day-old biofilms of (*A*) the WT ancestral strain, (*B*) SCV, (*C*) MCV, and (*D*) TCV were visualized on a Leica TCS SP5 confocal laser scanning microscope using *Bac*Light live/dead stain. Green cells indicate live cells and red cells indicate dead cells. Z-Scans were performed every 0.3 µm on each field of view. White bars represent 25 µm.

**Fig. 4.—:**
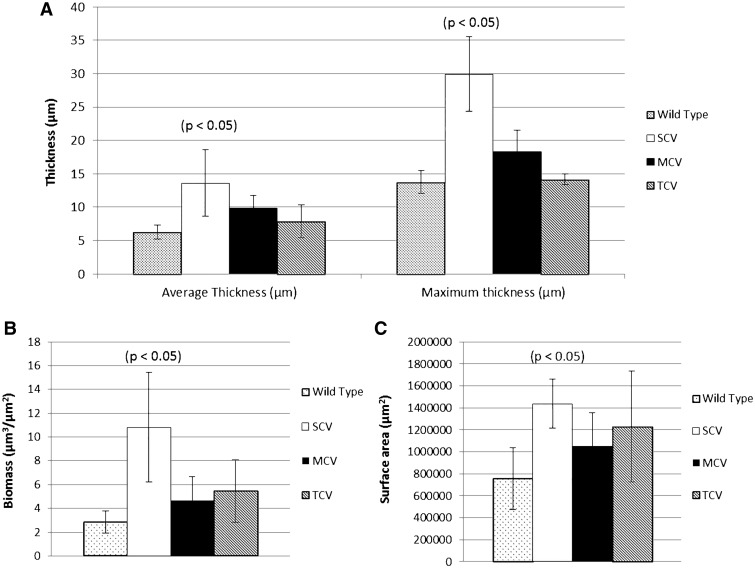
COMSTAT analysis of biofilm-derived colony variants. Biofilm formation of the biofilm-derived variants quantified using COMSTAT 1 and the program Matlab (R2012a) (Heydorn et al. 2000) for triplicate 3-day-old biofilms of each variant type, grown in MatTek culture plates under static conditions. Graph depicts (*A*) the mean average thickness and maximum thickness, (*B*) mean biomass, and (*C*) mean surface area. Error bars represent 95% confidence intervals.

Biofilm-derived colony variants were assessed for their ability to form biofilms compared to the ancestral strain. Confocal laser scanning microscopy (CLSM) revealed that the WT ancestor produced relatively flat biofilms with an average thickness of approximately 6 µm and small microcolony towers with maximum thickness of 12–15 µm (fig. 3A). In contrast, SCVs produced structured biofilms with an average thickness of approximately 13 µm and well-defined microcolony towers with a maximum thickness of 25–35 µm (fig. 3B). The two-sample *t*-test revealed that SCV biofilms had significantly greater biomass (µm^3^/µm^2^) (*T* = 3.26, df = 4, *P* < 0.05), surface area (µm^2^) (*T* = 3.56, df = 4, *P* < 0.05), average thickness (µm) (*T* = 2.81, df = 4, *P* < 0.05), and maximum thickness (µm) (*T* = 5.47, df = 4, *P* < 0.01) compared to the ancestral strain (Fig. 4). MCVs produced slightly larger biofilms than WT with an average thickness of approximately 10 µm and maximum thickness of 15–20 µm (fig. 3C). TCVs produced biofilm comparable with WT; with an average thickness of 8 µm and small microcolony towers with a maximum thickness of 13–14 µm (fig. 3D). Biofilms produced by the MCVs and TCVs did not differ significantly from WT. The percentage of dead cells within the biofilms was not significantly different between variants.

**Fig. 5.—:**
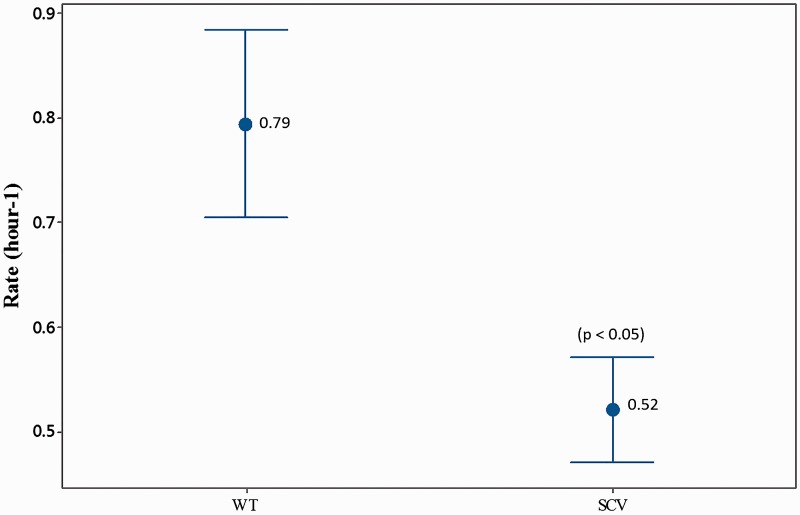
Mean growth rate of biofilm-derived SCV phenotype. The growth rate was assessed for SCVs from the exponential growth phase (between 4 and 8 h of growth) from triplicate growth experiments. SCV rate was calculated using pooled data from all 12 sequenced SCVs. A two-sample *t*-test was used to compare SCV rate to the WT rate. Numbers signify mean values. Error bars represent 95% confidence intervals.

**Fig. 6.—:**
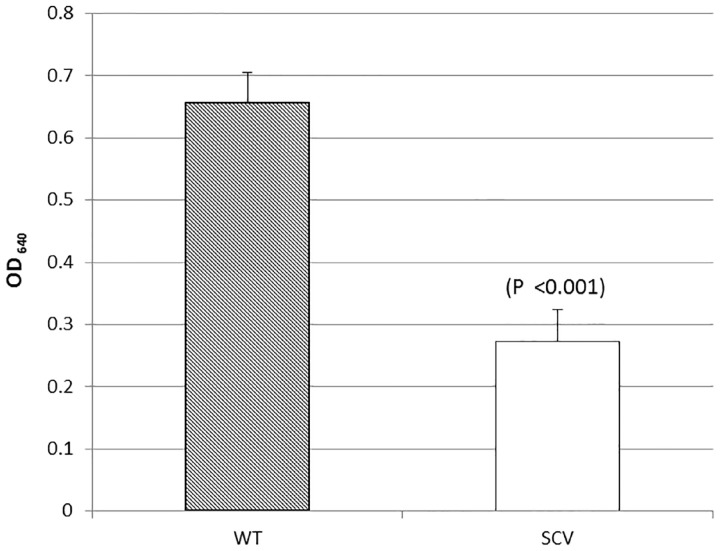
Capsule quantification of the SCV phenotype. Capsule quantification was determined by staining the pneumococcal capsule with Stains-all solution. The absorbance was measured at optical density 640 nm and subtracted from the negative control. Capsule quantification was calculated using pooled data from all 12 sequenced SCVs; graph depicts mean absorbance. Error bars represent 95% confidence intervals. Graph represents pooled data from all 12 sequenced SCVs.

Phenotypic profiling using the API Rapid ID 32 Strep assay confirmed that all SCVs were *S. pneumoniae* and not contaminants. Profiling also revealed that 8 of the 12 SCVs were unable to metabolize one or all of the following carbon substrates: D-trehalose, pullulan, maltose, and D-saccharose. Furthermore, SCVs exhibited a reduced growth rate compared to the WT (*T* = 2.84, df = 37, *P* < 0.01) (fig. 5) and a decrease in capsule staining compared to the WT (*T* = 7.79, df = 40, *P* <0.001) (Fig. 6).

To identify mutations responsible for the phenotypic diversity observed in the SCVs, a total of 12 SCVs were harvested from a 3-, 6-, or 9-day biofilm from two independent biofilm experiments (five SCVs from experiment 1 and 7 SCVs from experiment 2) (table 1). The 12 SCVs were sequenced using next-generation sequencing and compared to the 22F ancestral strain. Table 1 lists the confirmed mutations identified in this work. Notably all sequenced SCVs were shown to contain independent mutations within the DNA-directed RNA polymerase delta subunit (*rpo*E). With the exception of SCV5D3E1 and SCV7D9E1, these mutations were at different positions within the gene; SCV5D3E1 and SCV7D9E1 were isolated from separate biofilms, thus also occurred independently. Of the 12 SCVs sequenced, seven SCVs contained single-nucleotide substitutions resulting in an early stop codon, one SCV had an insertion mutation resulting in a coding frameshift, and four SCVs contained large-scale deletions ranging from 51 to 264 bp (table 1). Polymerase chain reaction (PCR) of the *rpo*E gene was employed to confirm the deletions seen in the whole-genome analysis. Sequence analysis identified short homologous regions flanking the deletions in three SCVs (SCV9D9E1, SCV1D3E2, and SCV2D6E2), suggesting underlying recombination events; there was no evidence to suggest that the deletion in SCV7D9E2 was a result of a recombination event. No change in the multilocus sequence typing (MLST) profile of the SCVs was observed.

**Table 1:** **SCV Mutations Identified Using Next-Generation Sequencing**

Variant	Reference Position	WT	Variant	Gene Description	Effect	Coverage	Percentage Confidence
SCV1D3E1	423010	G	T	DNA-directed RNA polymerase delta subunit	E173Stop	49×	98
SCV5D3E1	422977	G	T	DNA-directed RNA polymerase delta subunit	E163Stop	69×	100
SCV3D9E1	328131	A	C	Choline binding protein J	Q118P	30×	96.7
SCV3D9E1	422921	C	A	DNA-directed RNA polymerase delta subunit	S144Stop	44×	100
SCV3D9E1	908199	T	G	Hydrolase, putative	L26V	27×	96.4
SCV7D9E1	422977	G	T	DNA-directed RNA polymerase delta subunit	E163Stop	243×	100
SCV9D9E1	422795–423059	A	—	DNA-directed RNA polymerase delta subunit	Deletion	NA	NA
SCV9D9E1	1620361	C	A	Iron compound ABC uptake transporter permease protein PiuB	A278D	47×	97.4
SCV1D3E2	422765–422894	A	—	DNA-directed RNA polymerase delta subunit	Deletion	NA	NA
SCV2D6E2	422799–423063	G	—	DNA-directed RNA polymerase delta subunit	Deletion	NA	NA
SCV4D6E2	422947	G	T	DNA-directed RNA polymerase delta subunit	E153Stop	197×	99.5
SCV4D6E2	591574	G	T	Beta-galactosidase	A146S	129×	98.5
SCV4D6E2	1396435	C	T	Glycine/D-amino acid oxidases family	R412H	137×	100
SCV6D6E2	423020	—	G	DNA-directed RNA polymerase delta subunit	Insertion	92×	93.9
SCV5D9E2	423028	G	T	DNA-directed RNA polymerase delta subunit	E180Stop	146×	100
SCV6D9E2	422962	G	T	DNA-directed RNA polymerase delta subunit	E158Stop	122×	99.2
SCV7D9E2	423028–423090	G	—	DNA-directed RNA polymerase delta subunit	Deletion	NA	NA
SCV7D9E2	1035274	G	A	Streptococcal histidine triad protein	Q111Stop	106×	100

Note.—D3, day 3 biofilm; D6, day 6 Biofilm; D9, day 9 biofilm, E1, experiment 1; E2, experiment 2.

## Discussion

Increasing evidence suggests that carriage of the pneumococcus in the nasopharynx of humans is mediated by biofilm formation (Marks et al. 2012; Blanchette-Cain et al. 2013). This work used conventional phenotyping and whole-genome sequencing to characterize the genetic diversity that arises among biofilm-derived pneumococci. Three distinct colony morphologies were observed after 3 days of growth, including a small colony phenotype. This variation in colony morphology is consistent with previous studies involving other pneumococcal serotypes (Allegrucci and Sauer 2007, 2008) suggesting that this phenomenon is not an isolated occurrence. Whole-genome sequencing revealed that all SCVs contained mutations within the DNA-directed RNA polymerase subunit gene (RpoE), including one insertion, seven substitutions, and four large-scale deletions. In all cases, these mutations would result in truncation of the *rpo*E gene product affecting the low complexity regions of the protein which have been linked to the specificity of DNA binding. Despite few SCV mutations overall, these parallel evolutionary events suggest that a strong selection pressure for *rpo*E-targeted mutations occurs within *S. pneumoniae* biofilms and may be important for biofilm development.

Of the four deletion events observed in this work, three are likely to be due to a single intramolecular recombination event. In all four deletion events, the first 8 bp of the deleted sequence commenced with the sequence pattern GA(C/A)GA(A/C)GA. This sequence pattern may be analogous to the *Chi* site seen in *Escherichia coli* (Prudhomme et al. 2002) and represent a recognition site for the pneumococcal recombinase machinery which in turn results in the deletions seen; however, this hypothesis remains to be tested. Eight of the 12 sequenced SCVs contained a mutation only in the *rpo*E gene with no other additional changes to the genome. With the exception of SCV5D3E1 and SCV7D9E1, all mutations occurred at different positions in the gene. This observation supports the hypothesis that mutations in the *rpo*E gene are directly linked with the SCV phenotype. The consistent high frequency of mutations in a single gene, in multiple replicates of independent experiments, is clear evidence of biofilm-mediated parallel evolutionary events in real time. Parallel evolution of specific genes has been shown to be clinically relevant in determining the pathogenesis of *Burkholderia dolosa* (Lieberman et al. 2011) and *P. fluorescens* (Kim et al. 2014). Such a phenomenon has also been reported previously in *Pseudomonas aeruginosa* (McElroy et al. 2014); however, this study presents some of the best evidence of parallel evolutionary events in *S. pneumoniae*.

To date, RpoE has not been studied in *S. pneumoniae.* Using *Bacillus subtilis* as a model organism, RpoE has been attributed to increasing transcriptional specificity by increasing binding of RNAP to promoter sequences (Achberger and Whiteley 1981; Achberger et al. 1982). Additionally, RpoE has been attributed to increased efficiency of RNA synthesis and decreased affinity for nucleic acids due to enhanced recycling of RNA (Juang and Helmann 1994; Lopez de Saro et al. 1999). Understanding of the RpoE function is based on gene deletion studies; *rpo*E deletion mutants have been shown to be viable suggesting that RpoE is nonessential (Lopez de Saro et al. 1999); however, phenotypically *rpo*E mutant strains have be shown to display increased lag phase, altered cell morphology (Lopez de Saro et al. 1999), and altered biofilm architecture (Xue et al. 2010). In all studies to date, disruption of the *rpo*E gene has been mediated by deletion or alteration of the sequence (Lopez de Saro et al. 1995; Xue et al. 2010; Weiss et al. 2014). Our experiments provide evidence that mutations affecting the *rpo*E gene occur spontaneously during biofilm development. Their recurrence suggests an advantage in terms of growth and/or survival within biofilms.

*Streptococcus pneumoniae* is a nutritionally fastidious facultative anaerobe, but the availability of a glucose source within the human nasopharynx is thought to be in low concentrations (Philips et al. 2003). As biofilm formation is thought to represent a survival strategy in a nutritionally limited environment (Domenech et al. 2012), possibly, the reduction in carbon substrate utilization on the API strip may reflect selection for strains with reduced metabolic capacity and growth rate because they are better adapted to survive under biofilm conditions. Alternatively, there may be selection against the maintenance of energetically costly pathways not required under biofilm conditions. Further experimentation would be required to determine whether either of these hypotheses are correct.

We hypothesize that the mutations seen here can benefit the survival of *S. pneumoniae* in biofilms by altering gene expression in favor of colonization rather than virulence. In Gram-positive bacteria, RpoE has been shown to be important for rapid changes in gene expression to adapt to changing environmental conditions (Xue et al. 2010; Xue et al. 2011; Xue et al. 2012; Rabatinova et al. 2013). The reduction in growth rate seen in SCVs in this study is consistent with RpoE studies in *B. subtilis* (Lopez de Saro et al. 1999). The slower growth that we observed may be beneficial to a subpopulation of SCVs in the biofilm because it may allow bacteria to avoid direct competition between neighboring cells under conditions of limited nutrient availability. A number of studies have shown biofilm variants to have reduced virulence (Sanchez et al. 2011), which may similarly reflect a change in gene expression that promotes survival within biofilms. Interestingly, RpoE has been shown to be upregulated in response to quorum sensing molecule autoinducer-2 in *Streptococcus mutans* (Sztajer et al. 2008).

Of note, additional point mutations were also observed in choline binding protein J, the streptococcal histidine triad protein, and the iron compound ABC uptake transporter permease protein (PiuB). This observation is relevant as mutations within cell surface proteins may inform future protein-based pneumococcal vaccine studies. Protein-based pneumococcal vaccine design should be cautious not to target cell surface proteins, which have a tendency to mutate, as this may result in vaccine-escape. It is interesting to note that we observed no mutations within the capsule genes of the biofilm-derived variants. This differs from previous studies that implicated a 7 kb deletion in the *cps*3DSU operon in the formation of SCV variants in serotype 3 (Allegrucci and Sauer 2007). Whether biofilm-mediated mutations are a result of natural selection acting on random mutational events or a process of targeted or adaptive mutation remains to be determined (Banas et al. 2007). In this work, the lack of SCV phenotypes in the planktonic population and the variety of *rpo*E mutations seen would suggest a strong selection pressure for *rpo*E-targeted mutations occurs within *S. pneumoniae* biofilms and may be important for biofilm development.

This work has shown that diversification under biofilm conditions resulted in the emergence of SCVs with parallel mutations within the *rpo*E gene. Further work is investigating the transcriptional and translational effect of *rpo*E mutation on biofilm development and its clinical significance, as well as comparisons of genomic diversification between disease and carriage isolates of *S. pneumoniae* 22F. This study presents an unprecedented level of biofilm-mediated parallel evolution in *S. pneumoniae* and suggests a strong positive selection for mutations in *rpo*E, which may identify *rpo*E as an important gene for biofilm formation, carriage, and pathogenicity of the pneumococcus.

## Materials and Methods

### Bacterial Strains

The clinical isolate (ID: sp_3016) of *S. pneumoniae* 22F (ST433) was obtained from the third year (October 2009–March 2010) of an on-going nasopharyngeal carriage study in children aged 4 years and under at University Hospital Southampton, UK. Isolates were stored at −80 °C in glycerol stock consisting of 50% BHI broth (Oxoid) and 50% glycerol (Sigma). Capsular serotyping of the reference isolate was performed using genomic DNA and multiplex PCR (Pai et al. 2006) and MLST was performed using Qiagen Genomic Services and ST’s assigned using the MLST website (www.mlst.net, last accessed 2012) prior to biofilm experimentation.

### Growth Curves

Isolates were grown on Columbia blood agar (CBA) plates (Oxoid) overnight, colonies were used to inoculate 10 ml of BHI broth, and subsequently incubated at 37 °C with 5% CO_2_ for 10 h. The optical density (OD_600_) was measured every hour and 100 µl of culture taken every 2 h to perform viable counts. Growth curves for each strain were performed in triplicate on separate days.

### Biofilm Culture and Colony Variation

1 × 10^8^ CFU/ml was inoculated into MatTek culture plates (MatTek Corporation Ashland, MA) or six-well micotitre plates (Nunc) were inoculated using 1 × 10^8^ CFU/ml as described previously (Hall-Stoodley et al. 2008). Triplicate biofilms were grown under static conditions at 37 °C with 5% CO_2_ for 1, 3, 6, and 9 days in two independent experiments. Half of the culture media was removed daily and replaced with equal quantities of fresh 1:5 BHI prewarmed to 37 °C. At each time point, biofilms were washed twice with fresh prewarmed 1:5 BHI, and the biomass was harvested using a cell scraper, vortexed, diluted to 10 ^−^^6^ and 100 µl was plated onto CBA (Oxoid) in triplicate to assess changes in colony morphology compared to the inoculum. The growth rate was assessed for SCVs using the equation µ = ((log10 *N* – log10 *N*0) 2.303)/(*t* − *t*0) and CFU data from the exponential growth phase (between 4 and 8 h of growth) from triplicate growth experiments.

### Assessment of Colony Morphology

Colony morphology was assessed based on size and regularity of the colony surface. Variants were defined as follows; SCVs (<0.5 mm), MCVs (0.5–1 mm), and TCVs (>1 mm). Colonies were visualized using a Leica MZ 16F stereomicroscope, and images were taken using a Leica digital camera with microscope and a 5 mm graticule scale. Colony diameters were quantified using the ImageJ analysis software (http://rsbweb.nih.gov/ij/, last accessed July 2012). A total of nine colony variants from triplicate biofilms were measured. Per experiment, up to 12 phenotypic variants of each morphology type were selected, per time point, from the triplicate biofilms. Variants were subcultured and stored at −80 °C in glycerol stock for future phenotypic and genetic analysis. Additional phenotypic characterization and whole-genome sequencing were performed on 12 SCVs harvested from two independent experiments. Five SCVs were characterized from experiment 1 (3 × 3-day-old biofilms and 2 × 9-day-old biofilms) and seven SCVs from experiment 2 (1 × 3-day-old biofilms, 3 × 6-day-old biofilms and 3 × 9-day-old biofilms).

### Visualization of the Biofilm

Biofilms were imaged using CLSM and *Bac*Light live/dead stain (Life Sciences). Triplicate biofilms of each variant type were cultured in MatTek culture plates (MatTek Corp., Ashland, OR) under static biofilm conditions for 3 days, washed twice with fresh warmed 1:5 strength BHI, and then stained according to the manufacturer’s instructions. All images were taken using a 63× objective on a Leica TCS SP5 confocal laser scanning microscope on a Leica DMI6000 inverted microscope frame. Five fields of view were taken per triplicate biofilm. Z-Scans were performed every 0.3 µm on each field of view. Biofilm formation was quantified using COMSTAT 1 and the program Matlab (R2012a) (Heydorn et al. 2000) to determine the average thickness and maximum thickness, total biomass, and surface area of the WT and SCV biofilms.

### API Rapid ID 32 Strep Assay

API Rapid ID 32 Strep assay (bioMérieux) was performed according to the manufacturer’s instructions.

### Capsule Quantification

Quantification of the capsule was adapted and performed according to Hammerschidt et al. (2005) and Rukke et al. (2012). SCVs were grown on CBA plates (Oxoid) overnight at 37 °C and 5% CO_2_. The growth was transferred to 10 ml of BHI and cultured to an OD equating to approximately 10^8^ CFU/ml (OD_600_ ∼ 0.3–4). Cultures (5 ml) were centrifuged (2500 × g at 4 °C for 10 min) to pellet the cells. The supernatant was discarded, the pellet washed twice in 0.5 ml phosphate-buffered saline, and then resuspended into 5 ml of sterile distilled water. Resuspended cells (250 µl) were transferred to a sterile microcentrifuge tube and 1 ml of stain-all solution (20 mg Stains-all [Sigma-Aldrich], 60 µl glacial acetic acid in 100 ml of 50% formamide) added. Absorbance was measured at 640 nm and subtracted from the negative control (250 µl of sterile distilled water stained with 1 ml of Stains-all). Capsule quantification was measured in triplicate, with each measurement performed on separate days.

### Genomic DNA Extraction

Genomic DNA for whole-genome sequencing was extracted from 10 ml cultures of BHI containing approximately 10^9^ CFU/ml using Qiagen Genomic tip 100/G in accordance with the manufacturer’s instruction for Gram-positive bacteria. Genomic DNA for PCR was extracted using the QIAamp DNA mini kit (QIAGEN) according to the manufacturer’s instructions with minor modifications. Frozen stocks of each isolate were streaked onto CBA (Oxoid) and incubated at 37 °C in 5% CO_2_. Colony growth was transferred using a sterile swab to 200 µl of lysis buffer (10 mM Tris pH8, 100 mM ethylenediaminetetraacetic acid pH8, 0.5% w/v sodium dodecyl sulphate) and incubated at 37 °C for 1 h. Proteinase K (20 µl) was added and samples were incubated at 56 °C for 1 h. The following steps were performed as described in the Qiagen QIAamp mini kit protocol.

### Whole-Genome Sequencing

Biofilm-derived variants and the ancestral WT 22F strain underwent shot gun paired end whole-genome sequencing using the Roche GS Junior and/or the Illumina MiSeq. The ancestral 22F strain was sequenced both on the Roche GS junior 454 sequencer and on the Illumina MiSeq using the 150 base, pair-end protocol. The 12 SCV samples were sequenced on the Illumina MiSeq (2 × 150 or 2 × 250 bp). For Roche GS junior 454 paired-end sequencing, genomic DNA was sheared and GS Titanium Library Paired End Adaptors were added to the fragments, emPCR was performed using the Roche GS Junior Titanium emPCR Kit (Lib L). Samples were loaded onto the Roche GS Junior PicoTiterPlate according to the manufacturer’s instructions and the Roche GS Junior Titanium Sequencing Kit. For Illumina MiSeq sequencing, all samples were sheared and libraries were prepared using the Nextera library prep kit, Illumina MiSeq reagent cartridge, and sequencing reagents were used according to the manufacturer’s instructions. Paired-end sequencing of the WT strain acted as the template for comparing the variants. Illumina sequence data were assembled de novo using Velvet (Zerbino 2010) and the Velvet optimizer script generating assemblies with an average coverage of 62×. Assemblies were assessed for quality using the assemblathon script, and satisfactory n50 scores were generated (mean = 40,945 bp) (Earl et al. 2011). For the ancestral 22F strain, Roche GS junior 454 sequence data were assembled de novo using Newbler GSassembler with Illumina sequence data to generate a more comprehensive reference sequence. The resultant assembly of the ancestral strain had an 86× depth of coverage. The de novo assembled scaffolds of the ancestral strain were aligned against strain D39 reference in Mauve (Darling et al. 2010) to arrange the scaffold. All gaps were removed to create a pseudoreference from which all annotations were based. Annotation of the ancestral strain de novo assembly was achieved using RAST, the online annotation service (Overbeek et al. 2014). Sequence data are available from the NCBI Sequence Read Archive (http://www.ncbi.nlm.nih.gov/sra/, last accessed March 2016) under the accession number: SRP071227.

### MLST of Biofilm-Derived Variants

All sequences were assembled de novo as described above; the assembled contigs were uploaded to the Centre for Genomic Epidemiology online service (Larsen et al. 2012) to assess for changes in MLST from the ancestral strain ST433.

### Mutation Analysis

Point mutations were identified by mapping variant genome sequences against the annotated ancestral strain reference. Illumina fastq reads were mapped against the ancestral 22F strain sequence using STAMPY (Lunter and Goodson 2011). The output sequence alignment mapping files were converted to binary alignment mapping files using SAMtools and variant discovery was performed using mpileup, bcftools, and gatk annotator (Li et al. 2009; McKenna et al. 2010) to generate variant call files (.vcf), which contained the list of nucleotide variants, their position, and quality metrics. Variants were filtered based on quality score (*q* value) and those with *q* values <20 were rejected. Alternatively, variant call files (.vcf) were generated using SNPtree1.1 (Leekitcharoenphon et al. 2012). Each mutation was manually curated by mapping the Illumina fastq files against the WT reference to generate bam files (.bam) and indexed bam files (.bai) using SAMtools. Bam files were subsequently visualized using Integrative Genomics Viewer (Robinson et al. 2011; Thorvaldsdottir et al. 2013) to determine coverage and percentage confidence for each variant. Furthermore, the percentage confidence of each mutation was calculated as the coverage of the identified variant base call divided by the total base call coverage at the variant position. Mutations underwent rigorous selection criteria to avoid mis-calling. Only variant positions that were present in both the mapping data and de novo assemblies (>20× coverage and a percentage confidence >90%) were classed as confidently identified mutations. Functional effects of the mutations were postulated using the ExPASy research portal (Artimo et al. 2012) and the EMBL SMART database (Schultz et al. 1998; Letunic et al. 2012).

### PCR of *rpo*E

To confirm the validity of the *rpo*E deletions in the SCVs, DNA from each SCV underwent PCR for the *rpo*E gene. The *rpo*E gene is 588 bp in length and primers were designed to amplify an amplicon of 609 bp, encompassing the full *rpo*E gene. Forward primer 5’-GAGGAGAAACGCTTTGGAATTAGAAG-3’ and reverse primer 5’-GCTAACTCTTATTCCTCGCTGGTTTC-3’ were designed. PCR program consisted of three stages: stage 1: 94 °C for 5 min; stage 2: 30 cycles of 94 °C for 30 s 50 °C for 30 s 72 °C for 30 s; stage 3: 72 °C for 10 min; and stage 4: pause at 4 °C. PCR products were run out on a 1% agarose gel containing GelRed for 45 min at 90 V and visualized under ultraviolet light. Bioline HyperLadder II was used to assess the size of the bands.

### Statistical Analysis

All statistical analysis was performed on Minitab 16.2 Statistical Software (www.minitab.com, last accessed November 2015). Normality of the data was determined using the Anderson–Darling test and equality of variance was determined using the *F* test. Normality and equal variance were achieved after square root transformation of the growth rate and capsule staining data. Prior to analysis, data from all 12 sequenced SCVs were pooled and the two-sample *t*-test was used to determine a significant difference between the SCV phenotype and the WT 22F strain.

### Ethics Statement

Ethical approval for the collection of nasopharyngeal swabs from children aged 4 years and under with written informed consent from parents/guardians was obtained from Southampton and South West Hampshire Research Ethics Committee ‘B’ (REC 06/Q1704/105). Ethical approval was not required for further use of the bacterial isolates from the swabs. 
